# A Systematic Review and Meta-Analysis of Systemic and Local Antibiotic Therapy in the Surgical Treatment of Peri-Implantitis

**DOI:** 10.3390/antibiotics12071223

**Published:** 2023-07-24

**Authors:** María Baus-Domínguez, Sara Bakkali, Paula Hermida-Cabrera, María-Angeles Serrera-Figallo, José-Luis Gutiérrez-Pérez, Daniel Torres-Lagares

**Affiliations:** 1 Departamento de Estomatología, Facultad de Odontología, Universidad de Sevilla, 41009 Sevilla, Spain; bakkali.sara03@gmail.com (S.B.); paulahermidacabrera@gmail.com (P.H.-C.); jlgp@us.es (J.-L.G.-P.); danieltl@us.es (D.T.-L.); 2 Hospital Universitario Virgen del Rocío, Universidad de Sevilla, 41013 Sevilla, Spain

**Keywords:** antibiotics, probing pocket depth, bleeding on probing, peri-implantitis, surgical treatment, resective therapy, regenerative therapy

## Abstract

(1) Background: Given the existing controversy regarding the use of antibiotics in the treatment of peri-implantitis, this systematic review and meta-analysis aim to ascertain how beneficial the role of systemic and local antibiotics is in peri-implant surgical therapy, considering the harmful effects that they represent and the abuse of antibiotics in terms of global health. (2) Methods: To determine the therapeutic efficacy of the administration of antibiotics in the surgical treatment of peri-implantitis in terms of probing pocket depth (PPD) and bleeding on probing (BoP), electronic and manual bibliographic searches were carried out in the Embase and PubMed databases, collecting data that related to before and after treatment. (3) Results: The adjunctive use of local antibiotics provides significant improvements in PPD (MD = 1.29; 95% CI: 0.56 to 2.02; *p* ≤ 0.0006; I^2^ = 0%) when compared with surgical treatment alone. No significant differences were found in the other subgroup; that is, the use of systemic antibiotics did not significantly improve PPD changes in the surgical treatment of peri-implantitis (MD = 0.40; 95% CI: −0.15 to 0.95; *p* = 0.15; I^2^ = 0). (4) Conclusions: The use of local antibiotics in the surgical treatment of peri-implantitis seems to offer treatment improvements in terms of PPD and BoP, unlike that observed with the use of systemic antibiotics. However, these results should be taken with caution as they also depend on the type of surgical technique used, whether regenerative or resective. More research is needed on this topic to understand the role of local and systemic antibiotics in the treatment of peri-implantitis.

## 1. Introduction

Peri-implantitis has been defined as a pathological entity associated with the formation of biofilm on the surface of dental implants, the main characteristics of which are chronicity, inflammation of the peri-implant tissues and the loss of supporting bone, the latter being the distinguishing feature from another pathological process that can be triggered after implant placement—mucositis—putting the longevity of the implant at risk [1,2].

Numerous therapeutic solutions have been proposed for this condition, aiming either to reduce inflammation or bacterial load or to resolve the bone defects caused [2]. Supportive periodontal therapy (SPT) has been established as essential for implant success as it reduces the likelihood of reinfection and progression of the pathology [3]. However, over the years, attempts have been made to determine the most appropriate treatment to ensure the resolution of peri-implant pathology (in combination with subsequent maintenance and SPT). In this way, these treatments can be divided into non-surgical and surgical solutions [2].

Surgical therapy is required in cases where the scope for non-surgical therapy is limited or has failed, which has been reported in the literature to be the norm in cases of significant bone defects (>5 mm) [4].

This therapy allows access to the lesion by lifting a flap, after which mechanical debridement, resective surgery, implantoplasty, or the placement of bone graft materials to alleviate the defect caused (regenerative surgery) can be performed [5]. These treatments can be conducted independently or in combination.

These surgical procedures have shown significant results in alleviating the bone defects caused by peri-implantitis [6,7], but given the bacterial origin of the condition, the need for antibacterial measures to optimize and maximize the success achieved with surgical treatment cannot be ignored [1].

The very nature of peri-implantitis is such that antibiotic therapy is proposed as an adjuvant treatment to surgical intervention. Several authors have thus sought to establish an action protocol that guarantees the resolution of the pathology [1,4,5].

Antibiotics can be applied locally on the surface of the implants or administered orally, that is, systemically. Therefore, and in view of the existing controversy over antibiotic resistance, which is a global health problem that has also been described in the field of peri-implant treatment (as reported by Carlos M. Ardilla et al. (2022) [8]), the question arises as to the extent to which this adjuvant treatment may be beneficial and whether it provides significant results, weighing its use against the already growing resistance in the population.

Authors such as Malen Oen et al. (2021) [9] and José Nart et al. (2018) [10] reflected that systemic antibiotic therapy could not be used as a standardized treatment protocol due to the lack of information on the etiology of periodontitis, which prevents the use of narrower spectrum antibiotics. In fact, there are comparative studies [11] with a three-year follow-up showing that, over the long-term, the benefit of systemic antibiotic therapy does not differ from those groups in which it has not been applied.

This is why increasingly more studies are considering the use of local antibiotics [12] combined, for example, with bone graft materials in regenerative therapy [10,13,14], obtaining up to 100% survival at one-year follow-up and avoiding the overall problem of resistance.

Given the controversy existing in the available literature, the question arises as to how beneficial the role of systemic and local antibiotics is in the surgical therapy of peri-implantitis, knowing that the abuse of antibiotics is a serious global health problem. In particular, taking into account that most prescribers authorize the use of antibiotics empirically without knowing how effective their use is and what consequences their abuse has.

## 2. Results

### 2.1. Search Results

An electronic search of the two databases was carried out in October and November 2022, resulting in 155 articles (115 from Embase and 40 from PubMed). After eliminating duplicates and reading titles and/or abstracts, a total of 23 articles were selected. Manual searching identified a further four articles.

**Figure 1:**
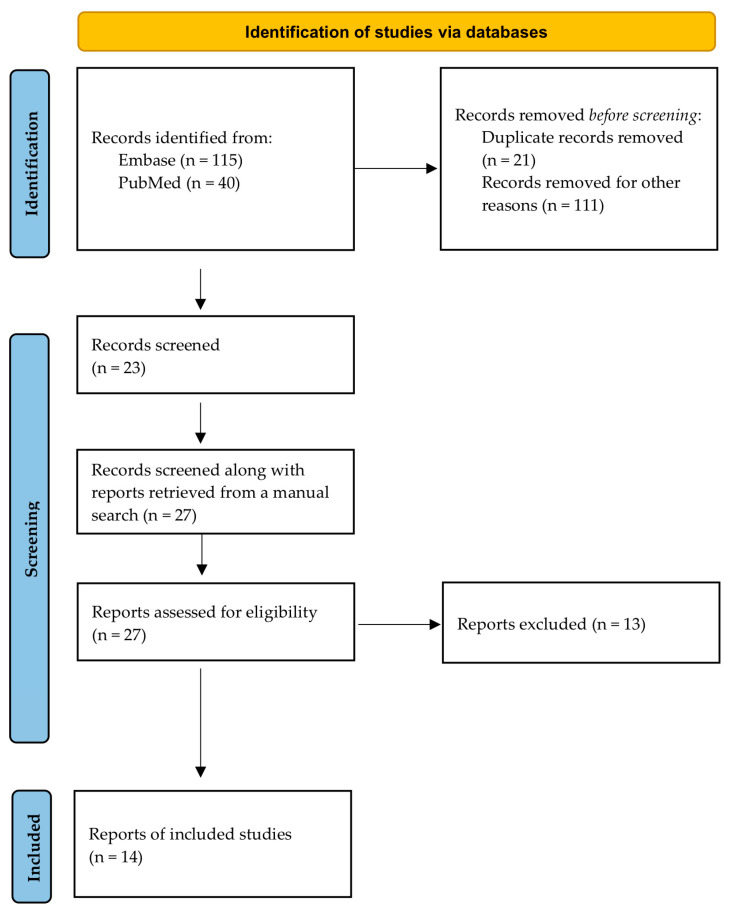
PRISMA 2020 flow diagram for new systematic reviews that included searches of databases.

Following a full-text review of all articles selected for inclusion, 13 manuscripts were excluded after their full reading. Thus, 14 articles were included in the final selection. Figure 1 shows the flowchart of the selection and inclusion method based on the recommendations of the PRISMA (Preferred Reporting Items for Systematic Reviews and Meta-Analyses) statement published in 2020. The data extracted for each article are shown in Table 1.

### 2.2. Quality Assessment and Risk of Bias

**Figure 2:**
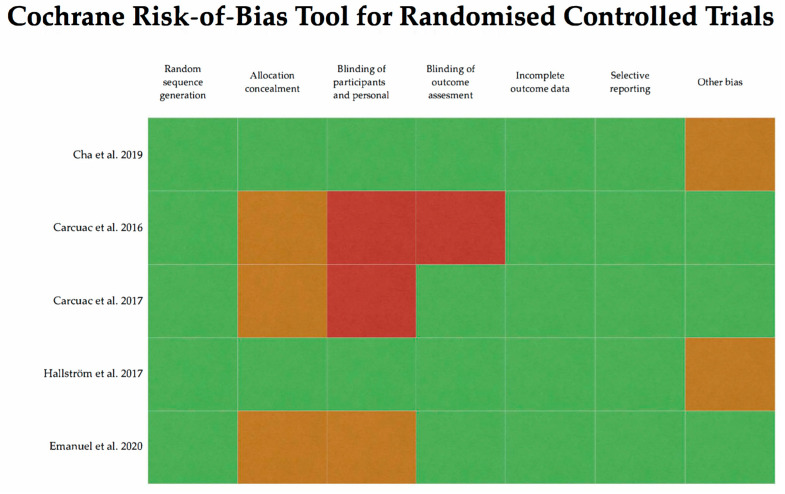
Quality and bias risk evaluation using Cochrane risk-of-bias tool for randomized controlled trials (Higgins et al., 2011 [15]). Papers [1,11,12,14,16] are considered as having a low (green), unclear (orange) or high (red) risk of bias.

**Figure 3:**
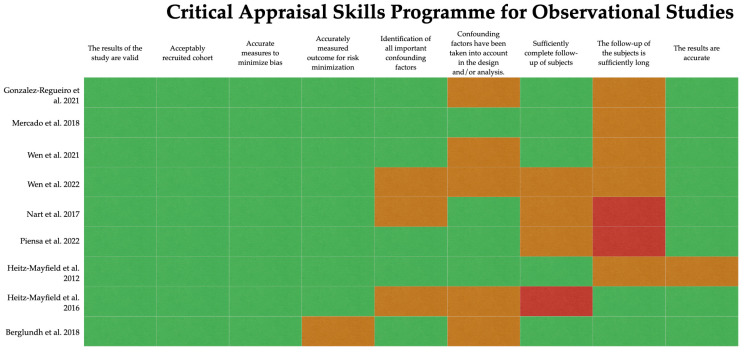
Quality evaluation of the non-RCTs using the Critical Appraisal Skills Program (CASP) (Zeng et al., 2015 [17]). The risk of bias in the included studies [10,13,18,19,20,21,22,23,24] was observed as low (green), unclear (orange) or high (red).

The assessment of the quality and risk of bias of the selected articles is summarized in Figure 2 and Figure 3. All studies selected are classified as having a low or moderate risk of bias.

### 2.3. Outcomes Regarding PPD

A total of 14 manuscripts (five randomized controlled trials (RCTs) and nine observational studies) analyzed the reduction in PPD and BoP of approximately 571 implants in 381 patients who had received surgical treatment for their peri-implantitis with or without local or systemic antibiotic therapy.

**Figure 4:**
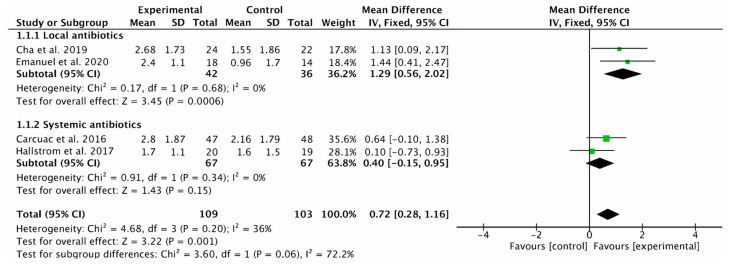
Probing pocket depth forest plot including only RCT (local antibiotics vs. systemic antibiotics) [1,12,14,16]. The weighted mean is presented at 95% CI. Heterogeneity was determined using Higgins (I^2^). A random-effects model was applied. Statistical significance was set at 0.05.

The adjunctive use of local antibiotic therapy provides significant improvements in PPD (MD = 1.29; 95% CI: 0.56 to 2.02; *p* ≤ 0.0006; I^2^ = 0%) when compared with surgical treatment alone. No significant differences were found in the other subgroup; that is, the use of systemic antibiotics did not significantly improve the PPD changes in the surgical treatment of peri-implantitis (MD = 0.40; 95% CI: −0.15 to 0.95; *p* = 0.15; I^2^ = 0) (Figure 4).

**Figure 5:**
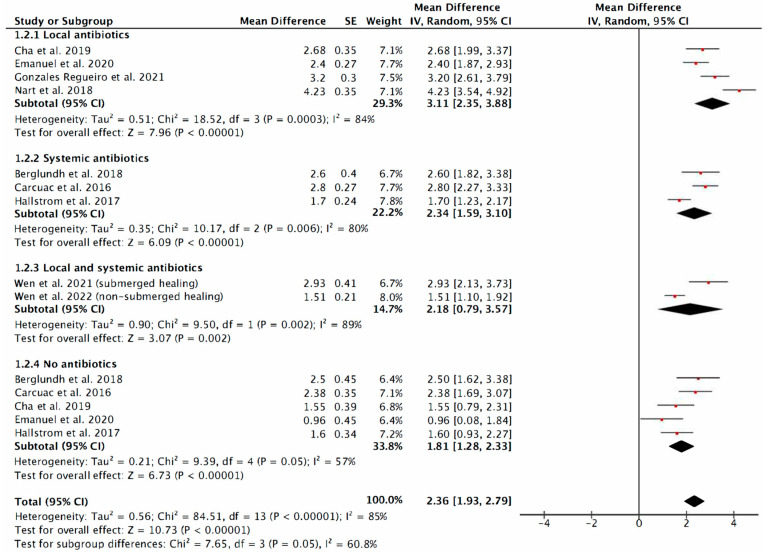
Probing pocket depth forest plot including all studies (local antibiotics vs. systemic antibiotics vs. local and systemic antibiotics vs. no antibiotics) [1,10,12,14,16,18,19,23,24]. The weighted mean is presented at 95% CI. Heterogeneity was determined using Higgins (I^2^). A random-effects model was applied. Statistical significance was set at 0.05.

The mean reduction in PPD when considering only the local antibiotics application was 3.11 mm, ranging from 2.35 to 3.88, and 2.34, varying from 1.59 to 3.10 when the systemic antibiotics prescription was considered. Surgical treatment without any antibiotics resulted in a reduction of 1.81 (95% CI: 1.28 to 2.33) (Figure 5).

### 2.4. Outcomes Regarding BoP

**Figure 6:**
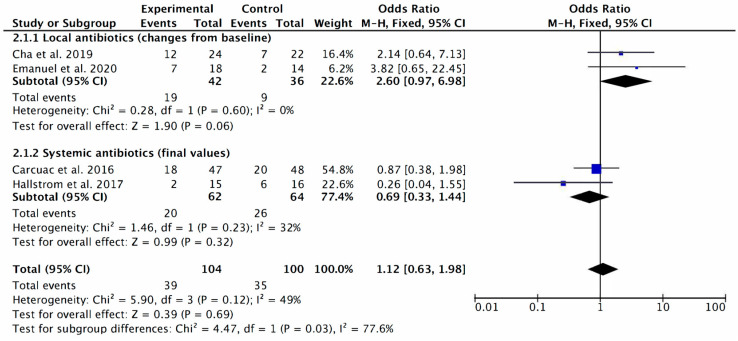
Bleeding on probing forest plot including only RCT (local antibiotics vs. systemic antibiotics) [1,12,14,16]. The weighted mean is presented at 95% CI. Heterogeneity was determined using Higgins (I^2^). A random-effects model was applied. Statistical significance was set at 0.05.

A positive association was found between the use of local antibiotics during the surgery and the reduction in BoP, but the magnitude of this association was small (odds ratio 2.66; 95% CI: 0.97 to 6.9) and almost statistically significant (*p* = 0.06). The systemic antibiotics administration did not result in lower odds of BoP (odds ratio 0.69; 95% CI: 0.33 to 1.44; *p* = 0.32; I^2^ = 0) (Figure 6).

## 3. Discussion

To date, this manuscript is the first systematic review of the effectiveness of the local and/or systemic application of antibiotics in the surgical treatment of peri-implantitis. This last point is important because, in this review, we only analyze the comparative results of the treatment of peri-implantitis using surgical techniques and do not analyze other, more conservative treatments.

The aim of this systematic review and meta-analysis was to determine the best available scientific evidence on the efficacy of local and/or systemic administration by comparing clinical parameters, including PPD and BoP, before and after the surgical treatment of peri-implantitis.

A recent systematic review warns of the indiscriminate use of antibiotics not only in the treatment of peri-implantitis but also in other surgical procedures with the aim of preventing early dental implant failure in healthy patients [8]. This same systematic review warns about increasing resistance to antimicrobials, especially metronidazole and clindamycin, by bacteria, such as *P. gingivalis*, *A. actinomycetemcomitans*, *F. nucleatum* and *P. intermedia/nigrescens*, as well as the great resistance of *Prevotella* spp., compared with penicillin, which is correlated with higher consumption of antibiotics by the investigated community [8]. Similarly, they report that most doctors empirically prescribe antibiotics without having performed a previous culture [8,10].

In terms of the local administration of antibiotics, this systematic review and meta-analysis includes five articles, of which [10,12,13,14,18] two were prospective studies, one was a case series, and two were randomized controlled clinical trials. However, only four of the five articles were used for quantitative analysis, excluding the article by Mercado et al. (2018) [13] for not specifying mean reduction. This makes a total of 138 implants studied. In terms of reducing PPD, the analysis of the RCTs shows a mean additional reduction of 1.29 mm (95% CI: 0.56 to 2.02; *p* ≤ 0.0006; I^2^ = 0%) (Figure 4), when comparing the experimental group treated with minocycline ointment by Cha et al. (2019) [12] or treated with D-PLEX_500_ (bone graft + doxycycline hyclate) by Emanuel et al. (2020) [14] versus those implants that did not receive local antibiotics treatment. The total PPD reduction including all studies, where the group effect was measured, was 3.11 mm (95% CI: 2.35 to 3.88; *p* ≤ 0.00001; I^2^ = 84%) (Figure 5).

Further elaborating on the results of the RCTs, Cha et al. (2019) [12] showed a mean test group reduction of 3.75 ± 1.30, starting from the deepest PPD 7.33 ± 2.33; and a control group reduction of 4.64 ± 1.55, starting from a shallower PPD (7.10 ± 1.20 mm) at 6 months post-surgery. The change in mean PPD between the baseline and 6 months was significantly greater in the test group than in the control group (2.68 ± 1.73 and 1.55 ± 1.86 mm, respectively, *p* = 0.039), where they also reported the absence of *P. gingivales* 6 months after applying the minocycline ointment. However, Emanuel et al. (2020) [14] did not observe statistically significant differences between the control and study groups at 6 months after evaluation, but did so at 12 months (−2.88 ± 1.52 (4.89 ± 1.32) vs. −1.64 ± 2.13 (6.07 ± 1.94)), where they also reported an important clinical significance due to the improvement in peri-implantitis lesions where D-PLEX_500_ was used.

On the other hand, cohort studies, such as that of Gonzalez-Regueiro et al. (2021) [18], where Implacure^®^ antibiotic solution and piperacillin/tazobactam were used for implant surface decontamination, showed a PPD reduction at 12 months of 3.20 ± 2.0 mm (*p* < 0.001), whereas that of Nart et al. (2018) [10], in which allograft was combined with vancomycin and tobramycin, reported the greatest reduction in PPD at 12 months of 4.23 ± 1.47 mm. Both studies had the limitation of the absence of a control group with which to compare their results. In the same way, as already reported by Cha et al. in their article, the variations in terms of PPD results will not only depend on the design of the implant, but also on the initial PPD of the defect [12].

It seems that the use of local antibiotics does offer an advantage over the surgical treatment of peri-implantitis, since the results reported in this meta-analysis are significant. However, the use of local antimicrobials is not without complications, since cases of resistance, alteration of the autochthonous microflora and hypersensitivity have been reported [8]. In this sense, a group of researchers reported that 71.7% of the patients with peri-implantitis studied were resistant to doxycycline, amoxicillin, metronidazole and/or clindamycin [10,18].

With regard to the systemic administration of antibiotics, this systematic review and meta-analysis does not show statistically significant results in terms of PPD reduction in implants treated using surgical techniques. Seven articles [1,11,16,20,21,22] were included for analysis, of which one was a retrospective case series, one was a retrospective clinical study, two were prospective clinical studies and three were randomized controlled clinical trials. However, only three of the seven articles were used for quantitative analysis, excluding the RCT by Carcuac et al. 2017 [11] since they worked with the same sample as in 2016 [1], and the case series by Pilenza 2022 [20] and two prospective clinical studies by Heitz-Mayfield et al. [21,22] because they did not specify mean reductions; thus, a total of 229 implants were studied. In terms of reducing PPD, the analysis of the RCTs shows a mean additional reduction of 0.40 mm (95% CI: −0.15 to 0.95; *p* = 0.15; I^2^ = 0) when comparing the experimental group treated with amoxicillin by Carcuac et al. (2016) [1] or azithromycin by Hallstrom et al. (2017) [16] versus those patients who did not receive systemic antibiotics treatment (Figure 4). However, if the results of the study by Carcuac et al. 2017 [11], where the follow-up was longer, little difference in PPD was observed between the groups (3.00 mm ± 2.24 in the experimental group and 2.38 mm ± 2.55 in the control group).

The total PPD reduction was 2.34 mm (95% CI: 1.59 to 3.10; *p* = 0.00001; I^2^ = 80), including in the analysis the observational study by Berglundh et al. (2018) [19] (Figure 5).

For their part, Carcuac et al. (2016) [1] showed the greatest reduction in PPD in this category (2.80 mm), with the surgical treatment used being a resective treatment with pocket removal, and the study group with the best results being the one treated with systemic antibiotics and using saline for the decontamination of the implant surface, whereby the local use of antiseptics did not seem to generate any positive synergistic effect on the treatment. In contrast, Hallstrom et al. [16] performed granulation tissue debridement with mucoperiosteal flap design, but did not specify resective pocket removal treatment. In turn, they stated that no significant improvements were observed in terms of PPD.

These results are in contrast with those reported in a series of prospective cases [25] in which they refer to a series of cases that claimed to observe promising results by combining mechanical debridement with systemic antibiotic therapy. This same prospective study, in which they performed a debridement of the implant surface in conjunction with a systemic administration of amoxicillin and metronidazole, observed a mean reduction in PPD of 2.14 mm; however, they are aware of its limitations, including the absence of a control group that made it impossible to evaluate the efficacy of the treatment [25]. Furthermore, a recent systematic review of the efficacy of metronidazole as an adjuvant in the therapy of peri-implantitis [26] warned that the combination of amoxicillin together with metronidazole should be limited to patients with specific microbiological profiles, as in the case of patients testing positive for *A. actinomycetemcomitans*. Since it is well known that most prescribers do not perform a bacteriological examination [8,27], in addition to the fact that combined management can contribute substantially to the advancement of antibiotic resistance, as already shown by many species [8], the routine use of this therapy arouses significant disagreement among researchers.

Along the same lines, Hallstrom et al. [16] also reported no significant improvements in terms of bone gain; however, Carcuac et al. [1] seem to have observed greater bone gain in the groups that received treatment with antibiotics. Nevertheless, the likelihood of successful treatment with adjuvant systemic antibiotics in patients with surface-modified implants was found to be low [1], a result that seems to be in line with that described in a systematic review [8] in which adverse results were reported for the mechanical management of peri-implantitis, while surgical therapy only resulted in a slight increase in bone level. For their part, Berglundh et al. [19] reported that the average PPD reduction and change in bone level at the last follow-up for all patients were 2.6 ± 2.2 mm and −0.1 ± 1.6 mm, respectively. Thus, the differences in terms of changes between those patients receiving antibiotic therapy after resective treatment and those not receiving it are small, with worse results observed in surface-modified implants.

One could come to the conclusion that the results of the studies that included systemic antibiotic therapy showed results that are not statistically significant due to the surgical technique used, since, in these cases, resective and non-regenerative techniques were reported. However, there is no reliable evidence to recommend the most effective intervention [28,29], although a recent systematic review and meta-analysis [28] reported that augmentation techniques appear to offer better results in terms of radiographic bone fill, but are comparable when it comes to reducing peri-implant soft tissue inflammation with respect to open flap debridement techniques. It is clear that this can affect the results in a global way, but it is not considered an obvious confounding factor, since the article by Cha et al. [12] where no bone graft was used offers statistically significant differences with respect to the use of local antibiotics. On the other hand, the bioavailability of antibiotics at the bone level should be considered, since, in the case of beta-lactam antibiotics, it is only between 10% and 20% of the serum concentrations reached, and this, together with the cases in which resistance is reported, would render useless the application of systemic antibiotics in the treatment of peri-implantitis [10].

With regard to the combined local and systemic administration of antibiotics, this systematic review and meta-analysis shows a statistically significant reduction in terms of PPD and BoP in implants treated with a regenerative surgical technique. Two articles [23,24], both prospective controlled clinical studies, were included for analysis, making a total of 59 implants studied. Both studies were conducted by the same research group, but on different patient samples. In the first study, Wen et al. (2021) [23] (30 implants), after defect measurements, performed a decontamination of the implant site with exposed coils by means of implantoplasty, followed by surface polishing with abrasive air and the application of 250 mg of tetracycline for 5 min. This was followed by reconstructive surgery via vertical bone regeneration using a dPTFE membrane together with 60% allograft, 20% xenograft and 20% autograft. All treated implants were submerged for 8 months. The post-operative instructions included the prescription of 500 mg of amoxicillin three times a day for 10 days, or 250 mg of Zithromax (six tablets in total), two tablets on the first day and then once a day thereafter until depleted. On the other hand, in the study by Wen et al. (2022) [24] (29 implants), where the same implant surface decontamination protocol (including the same local antibiotic dosage) and the same post-surgical antibiotic dosage were followed, the difference was then in the bone regeneration used. In this case, it was the same bone “cocktail” but a different membrane (resorbable collagen membrane), as the technique was non-submerged, and therefore a healing abutment would be in place for 8 months prior to re-entry to evaluate the treatment. Statistically significant changes in terms of bone gain and PPD reduction were observed in both studies, with mean implant PPD reduction scores of 2.93 ± 0.25 mm for Wen et al. (2021) and 1.51 ± 1.17 mm for Wen et al. (2022). Therefore, the total PPD reduction shown in our meta-analysis is 2.18 mm (95% CI: 0.79 to 3.57; *p* = 0.002; I^2^ = 89) (Figure 5). The studies also reported a significant reduction from the pre-surgical visit to the final check-up, from 100% to 36.6% for Wen et al. (2021) [23], and from 100% to 34.5% for Wen et al. (2022) [24].

Moreover, focusing now on bleeding on probing (BoP) reduction, comparing the local application of doxycycline (D-PLEX_500_ (PolyPid and MIS Implants Technologies Ltd.; PolyPid Ltd.; Petach Tikva, Israel)) with no antibiotic application in the study by Emmanuel et al. (2020) [14], and given the mean reduction in bleeding on probing of all probed implant sites, a reduction in BoP of 36.6% was observed in the group treated with local antibiotics and a reduction of 15.2% was observed in the group that underwent the surgical protocol only.

**Table 1:** Data referring to each of the articles included in the study focusing on the primary outcomes regarding BoP and PPD reduction in the surgical treatment of peri-implantitis when using systemic or local antibiotics.

Author	Study Design	Patients and Implants	Test Group	Control Group	Follow-Up	BoP Reduction in Test Group	BoP Reduction in Control Group	PPD Reduction in Test Group	PPD Reduction in Control Group
Heitz-Mayfield et al. 2012 [21]	Prospective clinical study	24 patients (36 implants)	Amoxicillin (500 mg) and metronidazole (400 mg) 3 times a day, for 7 days	None	12 months	% for sites 4 sites: 22% implants to 3% implants 3 sites: 28% implants to 8% implants 2 sites: 31% implants to 25% implants 1 site: 19% implants to 17% implants 0 sites: 57% (only after treatment) No average reduction specified	None	5.3 mm ± 1.8 to 2.9 mm ± 0.8 No average reduction specified	None
Heitz-Mayfield et al. 2016 [22]	Prospective clinical study	24 patients (36 implants)	Systemic amoxicillin (500 mg) and metronidazole (400 mg), 3 times a day for 7 days	None	5 years	% for sites (baseline vs. 5 years) 4 sites: 22% implants to 12.5% implants 3 sites: 28% implants to 4% implants 2 sites: 31% implants to 4% implants 1 site: 19% implants to 37.5% implants 0 sites: 57% (only after treatment) to 42% implants No average reduction specified	None	5.3 mm ± 1.8 (baseline) to 2.9 mm ± 0.8 (1 year) to 3.2 ± 1.0 (5 years) No average reduction specified	None
Carcuac et al. 2016 [1]	Randomised controlled clinical trial	100 patients (179 implants) Test group: 52 patients (93 implants) Control group: 48 patients (86 implants)	10 d systemic antibiotic regimen (amoxicillin 2 × 750 mg daily) commenced 3 d prior to surgery G1: surgical treatment + CHX + systemic antibiotic G2: surgical treatment + saline + systemic antibiotic	Chlorhexidine or saline solution G3: surgical treatment + CHX G4: surgical treatment + saline	12 months	G1: 18 (39.1) G2: 16 (34.8) No average reduction specified	G3: 20 (44.4) G4: 18 (51.4) No average reduction specified	G1: 7.85 ± 1.57 changes −2.80 ± 1.87 G2: 7.93 ± 1.50 changes −3.44 ± 1.66	G3: 7.79 ± 1.69 changes −2.16 ± 1.79 G4: 7.78 ± 1.25 changes −1.69 ± 2.22
Carcuac et al. 2017 [11]	Randomised controlled clinical trial	67 patients (121 implants) Antibiotics group: 68 implants No antibiotics: 53 implants	10 d systemic antibiotic regimen (amoxicillin 2 × 750 mg daily) commenced 3 d prior to surgery	No antibiotics	36 months	BoP (%): 1Y vs. 3Y 22% to 45% No average reduction specified	BoP (%): 1Y vs. 3Y 18% to 28% No average reduction specified	Probing depth changes (mm) baseline to 3Y −3.00 ± 2.24	Probing depth changes (mm) baseline to 3Y −2.38 ± 2.55
Hallström et al. 2017 [16]	Randomised controlled clinical trial	39 patients (only 1 implant per individual) Test group: 20 patients Control group: 19 patients	Zithromax^®^ 250 mg × 2 at the day of surgery, and 250 mg × 1 per day during four additional days	No antibiotics	12 months	100% to 12.4 ± 9.2% No average reduction specified	100% to 13.3 ± 11.1% No average reduction specified	5.7 ± 1.0 to 4.0 ± 1.1 Mean difference (reduction) in PPD values between baseline and month 12 in the test group was 1.7 mm (SD ± 1.1, 95% CI: 1.1, 2.3, *p* < 0.001).	5.8 ± 0.9 to 4.2 ± 1.5 The corresponding reduction in the control group was 1.6 mm (SD ± 1.5, 95% CI: 0.8, 2.4, *p* < 0.001)
Mercado et al. 2018 [13]	Prospective cohort study	30 patients (30 implants)	One capsule of Doxycycline 100 mg was added to BioOss (Geistlich, Switzerland)	None	36 months	100% to 20% No average reduction specified	None	8.9 mm ± 1.9 to 3.50 mm ± 0.50 No average reduction specified	None
Nart et al. 2018 [10]	Prospective case series study	13 patients (17 implants)	Vancomycin (OSTEOmycin V^®^, European Cell and Tissue Bank, Wels, Austria) + Tobramycin (OSTEOmycin T^®^, European Cell and Tissue Bank, Wels, Austria)	None	12 months	100% to 29.4% Mean reduction 70.6%	None	Distal: 7.88 ± 1.22 to 3.66 ± 0.59. Mesial: 6.48 ± 1.26 mm to 3.45 ± 0.43 PPDd and PPDm, which were significantly reduced (4.23 ± 1.47 mm, *p* = 0.001 and 3.03 ± 1.21 mm, *p* = 0.001, respectively)	None
Berglundh et al. 2018 [19]	Retrospective study	50 patients (95 implants) Test group: 36 patients Control group: 14 patients	10 days of amoxicillin 2 × 750 mg daily, commencing 3 days prior to surgery	No antibiotics	2–11 years	100% to 61.1% No average reduction specified	100% to 69.2% No average reduction specified	Antibiotic group: 7.2 ± 1.3 to 4.6 ± 1.9 PPD changes −2.6 ± 2.4	Non-antibiotic group: 7.0 ± 0.9 to 4.6 ± 1.3 PPD changes −2.5 ± 1.7
Cha et al. 2019 [12]	Randomised controlled clinical trial	50 patients (50 implants) Test group: 25 patients Control group: 25 patients	Minocycline ointment (Periocline; Sunstar)	Placebo	6 months	Reduction of BoP was observed in the test group (42.72% ± 34.97%)	No reduction in the control group (63.64% ± 37.48%)	PPD (mm) at deepest site at baseline 7.33 ± 2.33 mm to 3.75 ± 1.30 Mean changes 3.58 ± 2.32 Mean changes in the mean at 4 sites 2.68 ± 1.73	PPD (mm) at deepest site at baseline 7.10 ± 2.10 mm to 4.64 ± 1.55 mm 2.45 ± 2.13 Mean changes in the mean at 4 sites 1.55 ± 1.86
Emanuel et al. 2020 [14]	Randomised controlled clinical trial	27 patients (32 implants) Test group: 14 patients (18 implants) Control group: 13 patients (14 implants)	D-PLEX_500_ (local doxycycline)	No antibiotics	12 months	Average of all sites: 36.3%	Average of all sites: 15.2%	6.76 ± 1.74 to 4.36 ± 1.41 PPD changes −2.40 ± 1.16	6.39 ± 1.78 to 5.43 ± 1.92 PPD changes −0.96 ± 1.70
Gonzalez-Regueiro et al. 2021 [18]	Prospective case series study	43 patients (43 implants)	Piperacillin/tazobactam 100/12.5 + antibiotic solution (Implacure^®^ [medtech Dental AG, Switzerland])	None	15 days after surgery then at 1, 3, 6 and 12 months	From 43 (100%) to 6 (14%) BoP showed a reduction of 86% at patient level (*p* < 0.001)	None	From 6.4 mm ± 2.1 to 3.2 mm ± 1.0 Mean reduction of 3.2 ± 2.0 mm (*p* < 0.001)	None
Wen et al. 2021 [23]	Prospective study	22 patients (30 implants)	Tetracycline: 250 mg (locally delivered antimicrobial) + 500 mg of amoxicillin 3 times a day for 10 days or 250 mg of Zithromax 6 tablets, 2 to be taken on the first day, and once daily thereafter	None	8–12 months	100% to 36.6% Mean reduction 63.3%	None	5.81 ± 1.48 mm to 2.91± 1.11 Mean gain for the implant sites amounted to 2.93 ± 0.25 at 8 months	None
Wen et al. 2022 [24]	Prospective case series study	24 patients (29 implants)	Tetracycline: 250 mg (locally delivered antimicrobial) + systemic antibiotic prescriptions to be taken orally for 10 (500 mg amoxicillin every 8 h) or 5 days (6 Zithromax 250 mg tablets, 2 to be taken on the first day, and once daily thereafter	None	8–12 months	100% to 34.5% (12 months) Mean reduction 65.5%	None	4.73 ± 1.15 mm to 3.22 ± 1 mm Mean reduction 1.51 ± 1.17	None
Pilenza 2022 [20]	Retrospective case series study	11 patients (20 implants)	Eight-day antibiotic therapy using the van Winkelhoff cocktail (375 mg Amoxicillin and 250 mg Metronidazole 3×/day)	None	12 months	90% to 20% No average reduction specified	None	4.9 mm ± 1.35 to 2.7 mm ± 0.66 No average reduction specified	None

Considering the time factor, which is relevant for determining the long-term efficacy of a treatment, Berglundth et al. (2018) [19] conducted an 11-year retrospective study in which no significant difference was observed between the group that underwent the systemic antibiotic regimen of amoxicillin for 10 days at 2 × 750 mg/day starting 3 days before surgical treatment and the group that did not start this regimen, with a BoP reduction of 61.1% and 69.2%, respectively.

Heitz-Mayfield et al. conducted two retrospective clinical studies with the same characteristics and different follow-up times. They studied 24 patients (36 implants) who underwent surgical treatment of peri-implantitis and subsequent systemic antibiotic therapy with 500 mg of amoxicillin and 400 mg of metronidazole 3 times a day for 7 days, obtaining data on the number of implant sites bleeding on probing (0, 1, 2, 3, 4), with a follow-up of 12 months [21] and 5 years [22]. At 12 months, bleeding at four sites per implant was reduced from 22% to 3%, and at 5 years, the reduction was from 22% to 12.5%. The complete resolution of peri-implantitis understood as the absence of BoP was 57% at 12 months and 42% at 5 years.

Along the same lines, and with regard to BoP, after 12 months of follow-up, Hallström et al. (2017) [16], in their RCT, reported a significant reduction of 100% to 12.4 ± 9.2% in the group subjected to systemic antibiotics treatment with 250 mg × 2 of Zithromax after surgery and 250 mg × 1 per day for 4 days after surgery, and a BoP reduction in the group without an antibiotic regimen of 100% to 13.3 ± 11.1%, where no means were specified.

Looking at the forest plot (Figure 6) that collected the results of the different studies, dividing them into local application [12,14] and systemic administration [1,16] of antibiotic therapy, a positive but small association is observed between the use of local antibiotic therapy during surgery and a reduction in bleeding after catheterization (odds ratio: 2.66; 95% CI: 0.97 to 6.9; *p* = 0.06; I^2^: 0%). However, the systemic administration of antibiotics does not show significant results in terms of BoP reduction (odds ratio 0.69; 95% CI: 0.33 to 1.44; *p* = 0.32; I^2^ = 0).

According to the meta-analysis, the administration of local antibiotics resulted in significant improvements in terms of PPD and BoP in patients with peri-implantitis treated with a surgical approach. This result is in line with the improvements observed by Toledano et al. [30] who reported a reduction in BoP of a half and a mean reduction in PPD of 0.30 mm. However, given recent microbial resistance, the use of locally administered antibiotics should also be evaluated individually.

Nevertheless, systemic antibiotics use remains a controversial issue, as currently there are many reviews stating that systemic antibiotics do not improve the surgical treatment of peri-implantitis in terms of PPD or BoP [26,28], statements that are consistent with that observed in our systematic review and meta-analysis.

Although the results of this systematic review and meta-analysis report the benefits of the local and systemic administration of antibiotics in the surgical treatment of peri-implantitis in terms of improving PPD and BoP, it has not been shown to have lasting efficacy [27].

The main limitations of this systematic review and meta-analysis are the lack of correctly conducted randomized controlled clinical trials (RCTs), where, in addition, two of them present a high risk of bias, since two of them did not propose a double-blind study. Similarly, in this study we only worked with 5 RCTs, which made it necessary to add 9 observational studies, thus increasing the sample in which we work. We are aware that meta-analyses must be carried out fundamentally on RCTs, but observational studies are usually included when RCTs on a subject are scarce, as is the case for this issue. Observational studies, in general, were well planned, with the greatest bias being patient follow-up, even though extensive follow-up is needed to correctly assess the results of the local and/or systemic application of antibiotics, the consensus report of the European Federation of Periodontology recommended the evaluation of treatments for peri-implant disease for at least 6 to 12 months [12]. None of the articles used present less than 12 months of follow-up, with the exception of Cha et al. who presented a six-month follow-up. On the other hand, another limitation that could be raised is the fact that the efficacy of different antibiotics is compared, since each of them can generate different responses. However, it is not considered a major confounding factor in our results, since, in the case of local antibiotics, where different antimicrobials are compared, a significant improvement is observed in terms of PPD, and BoP of the study group compared to that of the study group. The control group, compared to the group of systemic antibiotics, where there was greater unification in terms of the type of antibiotic and dosage, said that no improvement was observed.

Taking into account what has been previously discussed, it is concluded that this meta-analysis presents a high degree of external validity, since its results can be applied in daily clinical practice, with periimplantitis being a frequent real problem in dental offices. According to this meta-analysis, systemic antibiotics do not appear to improve the results in terms of PPD or BoP of the surgical treatment of periimplantitis, unlike the use of local antibiotics, so dentists can treat this pathology based on the best scientific evidence available and reduce the indiscriminate use of antibiotics, which, as has been previously stated, contribute to a global health problem, bacterial resistance.

## 4. Materials and Methods

### 4.1. Protocol and Registration

The study protocol was prepared in consideration of the Preferred Reporting Items for Systematic Review and Meta-Analysis (PRISMA) statement, and the transparency of the review was increased using the PRISMA checklist. The developed protocol was previously registered and allocated the identification number CRD42023415954 in PROSPERO, the International Prospective Register of Systematic Reviews database, hosted by the National Institute for Health Research (NIHR), Centre for Reviews and Dissemination, University of York, York (UK) (www.crd.york.ac.uk/PROSPERO, accessed on 10 April 2023).

### 4.2. Focus Question

The focus question was designed according to the PICO question: What efficacy can we expect from the administration of local and systemic antibiotics in the surgical treatment of peri-implantitis, in terms of improvement in probing pocket depth (PPD) and bleeding on probing (BoP)?

The PICO elements were as follows:

- Population (P): Patients with peri-implantitis.
- Intervention (I): Surgical treatment of peri-implantitis together with the systemic or local administration of antibiotics in patients with pre- and post-surgical evaluation.
- Comparison (C): Surgical treatment of peri-implantitis without the systemic or local administration of antibiotics in patients with pre- and post-surgical evaluation.
- Outcome (O): Results showing changes in the clinical diagnostic parameters of peri-implant health, including PPD and BoP, before and after (at least 6 months) the surgical treatment of peri-implantitis.
- Study (S): Randomized controlled trials (RCTs) and observational studies (cohort and case–control studies, and case series).

### 4.3. Search Strategy

The PubMed and Embase databases were searched for publications in peer-reviewed journals.

Only publications that were written in English and published in the last 5 years (2018–2022) were selected.

Bibliographies of previously published systematic reviews, meta-analyses and literature reviews were examined for manuscripts of interest that were not included in the electronic search.

The following search strategy design was used for each database:

PubMed: ((Periimplantitis OR peri-implantitis [mh] OR periimplantitides OR peri-implantitides OR “peri-implant infection” OR “peri-implant disease” OR “periimplant disease” OR “peri-implant bone loss” OR “periimplant bone loss” OR “periimplant mucositis” OR “peri-implant mucositis” OR periimplant OR peri-implant) OR (“bone loss” OR “bone resorption” OR “bone defect”)) AND (antibiotic OR antibacterial OR anti-bacterial OR bacteriocidal OR bacteriocide OR antimicrobial OR anti-microbial) AND (surgical OR surgery) AND (therapeutic OR therapy OR therapies OR treatment).

Embase: (‘periimplantitis’/exp OR periimplantitis OR ‘peri implantitis’/exp OR ‘peri implantitis’ OR periimplantitides OR ‘peri implantitides’ OR ‘peri-implant infection’ OR ‘peri-implant disease’ OR ‘periimplant disease’ OR ‘peri-implant bone loss’ OR ‘periimplant bone loss’ OR ‘periimplant mucositis’/exp OR ‘periimplant mucositis’ OR ‘peri-implant mucositis’/exp OR ‘peri-implant mucositis’ OR periimplant OR ‘peri implant’ OR ‘bone loss’/exp OR ‘bone loss’ OR ‘bone resorption’/exp OR ‘bone resorption’ OR ‘bone defect’/exp OR ‘bone defect’) AND (‘antibiotic’/exp OR antibiotic OR ‘antibacterial’/exp OR antibacterial OR ‘anti bacterial’ OR bacteriocidal OR ‘bacteriocide’/exp OR bacteriocide OR ‘antimicrobial’/exp OR antimicrobial OR ‘anti microbial’) AND (‘surgical’ OR ‘surgery’/exp) AND (therapeutic OR ‘therapy’/exp OR therapy OR therapies OR ‘treatment’/exp OR treatment).

### 4.4. Inclusion and Exclusion Criteria for Studies

The publications included in the study meet the following inclusion criteria:

- Randomized controlled clinical trials or observational studies (cohort and case–control studies and case series) conducted on adult patients where at least 6 months of follow-up is reported.
- Studies that provide all the necessary data to establish a correct diagnosis of peri-implantitis.
- Studies that correctly explain the surgical technique performed and that indicate the name of the antibiotic and the regimen administered.
- Studies that provide all the necessary data for assessing the effectiveness and efficacy of the treatment by comparing the changes observed in the clinical parameters and always including PPD and BoP.

Exclusion criteria:

- Studies with less than 6 months follow-up.
- In vitro studies.
- Studies conducted on animals.
- Literature reviews.
- Systematic reviews.

### 4.5. Study Selection and Data Extraction

The electronic and manual literature searches were conducted by two independent reviewers (M.B.-D. and P.H.-C.) who also selected candidate studies for inclusion in the systematic review and meta-analysis by reviewing the titles and abstracts of the publications, working within the guidelines of the above inclusion and exclusion criteria.

Full-text articles were similarly examined independently by both reviewers to determine their eligibility. Any discrepancies that may have arisen between the two reviewers regarding the selection and inclusion of any manuscript were discussed until a consensus was reached, where a third reviewer (S.B.) was also involved.

The manuscripts that were excluded, as well as their reasons for exclusion, have been formally recorded.

The critical reading of articles and the risk of bias assessment were carried out using the Cochrane Collaboration tool for assessing the risk of bias in randomized trials [15] and the Critical Appraisal Skills Programmed (CASP) for cohort studies, case–control studies and case series [17].

The data extraction was performed in duplicate by two researchers (M.B.-D. and S.B.). The data extracted were authors and year of publication; study design; number of patients and implants; surgical treatment performed alone or in combination with antibiotics (type of antibiotic and dosage); follow-up period of the study; BoP reduction; and PPD reduction.

Similarly, data regarding plaque index, presence of suppuration, keratinized mucosa, observed bone loss, microbiological evaluation and adverse effects, before and after surgical treatment in combination or not with local or systemic antibiotics, were recorded.

### 4.6. Data Analyses

The Review Manager (RevMan) Version 5.3 statistical software (the Cochrane Collaboration, Copenhagen, Denmark) was used to perform meta-analyses of the effect of systemic and local antibiotics in the surgical treatment of peri-implantitis on PPD and BoP.

Randomized clinical trials were analyzed separately using weighted mean differences (WMD) and standard deviations for continuous outcomes, and odds ratio with 95% confidence intervals for dichotomous outcomes, to estimate the treatment effect. When combining all types of studies, the standard error was calculated, with the generic inverse-variance method used.

The fixed-effects model was employed, when appropriate, when two or more randomized clinical trials were included in any comparison. However, the random-effects model was used to pool studies of different designs.

Subgroup analyses were performed according to the type of antibiotic.

Studies without enough data for meta-analysis were evaluated qualitatively.

A forest plot was created to illustrate the effects of the various studies and the global estimation in the meta-analysis. Statistical significance was defined as *p* < 0.05.

## 5. Conclusions

The results of the present meta-analysis show that the adjuvant use of local antibiotics during the surgical treatment of peri-implantitis was significant in terms of PPD compared with systemic antibiotics.

However, these data should be interpreted with caution since they may depend on the type of peri-implantitis treatment, as well as its severity and the design of the implant to be treated.

More research is needed for more conclusive results.

## Data Availability

The developed protocol was previously registered and allocated the identification number CRD42023415954 in PROSPERO, the International Prospective Register of Systematic Reviews database, hosted by the National Institute for Health Research (NIHR), Centre for Reviews and Dissemination, University of York, York (UK) (www.crd.york.ac.uk/PROSPERO, accessed on 10 April 2023).
